# Organelle activity organized by the endoplasmic reticulum-mitochondria encounter structure -ERMES- is essential for *Podospora anserina* development

**DOI:** 10.15698/mic2025.09.860

**Published:** 2025-09-12

**Authors:** Melisa Álvarez-Sánchez, Matías Ramírez-Noguez, Beatriz Aguirre-López, Leonardo Peraza-Reyes

**Affiliations:** 1 Departamento de Bioquímica y Biología Estructural, Instituto de Fisiología Celular, Universidad Nacional Autónoma de México, Mexico City, Mexico.

**Keywords:** mitochondria, peroxisomes, endoplasmic reticulum, lipid droplets, organelle interactions, fungi, sexual development

## Abstract

Eucaryotic cell functioning and development depend on the concerted activity of its organelles. In the model fungus *Podospora anserina*, sexual development involves a dynamic regulation of mitochondria, peroxisomes and the endoplasmic reticulum (ER), suggesting that their activity during this process is coordinated. The ER-Mitochondria Encounter Structure (ERMES) is a tether complex composed of the ER protein Mmm1 and the mitochondrial proteins Mdm10, Mdm12 and Mdm34, which mediates membrane contact-site formation between these organelles. This complex also mediates interactions between mitochondria and peroxisomes. Here we analyzed the role of the ERMES complex during *P. anserina* development. By studying a thermosensitive *MDM10* mutant, we show that MDM10 is required for mitochondrial morphology and distribution, as well as for peroxisome membrane-remodeling and motility. We discovered that lipid droplets exhibit a subapical hyphal localization, which depends on MDM10. MDM10 is also required for ER shaping and dynamics, notably of the apical ER domains of the polarized-growing hyphal region, in a process that involves the activity of the protein YOP1. We also show that apical ER shaping involves a Spitzenkörper-associated membrane traffic, which implicates MDM10, and that the mycelial growth defect of *mdm10* mutants is exacerbated when the ER-shaping proteins YOP1 or RTN1 are loss. Finaly, we show that MMM1 is strictly required for mycelial growth and sexual development, suggesting that its activity is essential. Our results show that the activity of distinct organelles depends on the ERMES complex, and that the function of this complex is critical for *P. anserina* growth and development.

## Abbreviations

ER - endoplasmic reticulum,

ERMES - ER-mitochondria encounter structure.

## INTRODUCTION

Eukaryotic cells are composed of multiple membrane-bound organelles that establish a cooperative distribution of the different cell tasks. Organelles maintain dynamic interactions and crosstalk, which allow them to coordinate their activity to sustain cell functioning and development. Physical interactions between organelles are defined by membrane contact sites, which are established by tethering protein complexes 1. Membrane contact-sites facilitate the exchange of metabolites, membrane lipids, ions and proteins between organelles, and participate in organelle biogenesis and quality control. Interorganelle contacts also play primordial roles in multiple aspects of organelle dynamics, such as the regulation of organelle remodeling, abundance, transport and distribution 2,3,4.

Mitochondria and the endoplasmic reticulum (ER) are essential organelles whose activity in the cell is closely interrelated. Distinct tethering protein complexes mediate contact sites between these organelles across eukaryotes, which are critical for lipid and Ca^2+^ transfer, and act as signaling hubs that regulate bioenergetic metabolism, cell homeostasis and death 5,6,7,8. Mitochondria-ER contact sites play key roles in the regulation of the dynamics of mitochondria -including their fission, fusion, distribution and quality control 9,10,11- as well as of the synthesis and maintenance of their DNA (mtDNA) 12.

The ER-Mitochondria Encounter Structure (ERMES) constitutes a key tethering complex mediating the association between these organelles. This complex is composed of the mitochondrial outer membrane β-barrel protein Mdm10 linked to the ER protein Mmm1 via the peripheral mitochondrial protein Mdm34 and the cytosolic Mdm12 13,14. The ERMES complex is conserved in fungi, and several of its components are present in distinct protist groups 15,16. Likewise, an MMM1 homologue tethers ER to mitochondria in animals 17,18. The ERMES complex mediates phospholipid exchange between the ER and mitochondria 13,19,20,21, and is critical for mitochondrion morphology, segregation, and selective elimination by mitophagy, as well as for mtDNA maintenance, distribution and inheritance 22,23,24,25,26,27,28,29. In addition, peroxisomes often localize adjacent to ERMES sites 14,30, and associate with mitochondria through a physical interaction between Mdm34 and the peroxisome membrane protein Pex11 31. Overproduction of ERMES proteins induces the formation of peroxisome-mitochondrion contacts 32, while they decrease in ERMES mutants 33, supporting that the ERMES complex mediates the association of mitochondria with peroxisomes.

Sexual development in the model fungus *Podospora anserina*, involves precise regulation of the dynamics of mitochondria, peroxisomes and the ER (reviewed in 34). In this mycelial ascomycete, peroxisomes change their distribution, abundance, and morphology at defined sexual development stages, in correlation with the activity of the peroxisome protein import machinery that drives peroxisome biogenesis 35,36,37. In addition, sexual development implicates changes in mitochondrial arrangement, and involves the remodeling of mitochondria and peroxisomes by the fission proteins DNM1 and FIS1, which are important for meiotic differentiation 38. Moreover, the ER also undergoes a developmental remodeling along sexual development that implicates the ER membrane-shaping reticulon protein RTN1, which is required for meiotic spindle activity and nuclear segregation 39. These observations suggest that sexual development involves the concerted regulation of the dynamics of these organelles. However, the relevance of their interactions along this process is unknown.

In *P. anserina*, a mutation in *MDM10* gene -*mdm10-1-* was identified in a screen for genetic suppressors of a mutation resulting in premature death. The *mdm10-1* mutation is a missense mutation that changes a leucine into a proline 28 residues before the protein carboxyl end (*mdm10^L420P^*) 40. This mutation alters the lifespan of the fungus, affects aerial mycelium formation and ascospore (meiotic spore) germination. In addition, the *mdm10-1* mutation leads to a thermosensitive phenotype, where mycelial growth is moderately reduced at 27°C and severely affected at 35°C. Likewise, *mdm10-1* mutants display aberrant mitochondrial morphology, whose severity is aggravated at 35°C 40. The participation of the ERMES complex in ER and peroxisome dynamics in mycelial fungi has not been studied. Moreover, its contribution to sexual development has not been addressed. Here, we found that, in addition to mitochondria morphology, MDM10 is required for proper mitochondrial distribution. We describe that MDM10 dysfunction alters lipid-droplet distribution, ER shaping, and peroxisome morphology and motility. We show that the defects in ER shaping produced by *mdm10-1* involve the activity of the protein YOP1, and that mycelial growth defects of *mdm10-1* mutant are exacerbated when the ER-shaping proteins RTN1 or YOP1 are missing. Still, we did not detect defects in early Golgi arrangement in *mdm10-1* hyphae. Finaly, we show that MMM1 is strictly required both for vegetative growth and sexual development, consistent with an essential role in *P. anserina*. Our results show that the dynamics of distinct organelles depend on the ERMES complex, and that the function of this complex is critical for growth and development in this fungus.

## RESULTS

### Mitochondrial arrangement and distribution depend on MDM10

**Figure 1:**
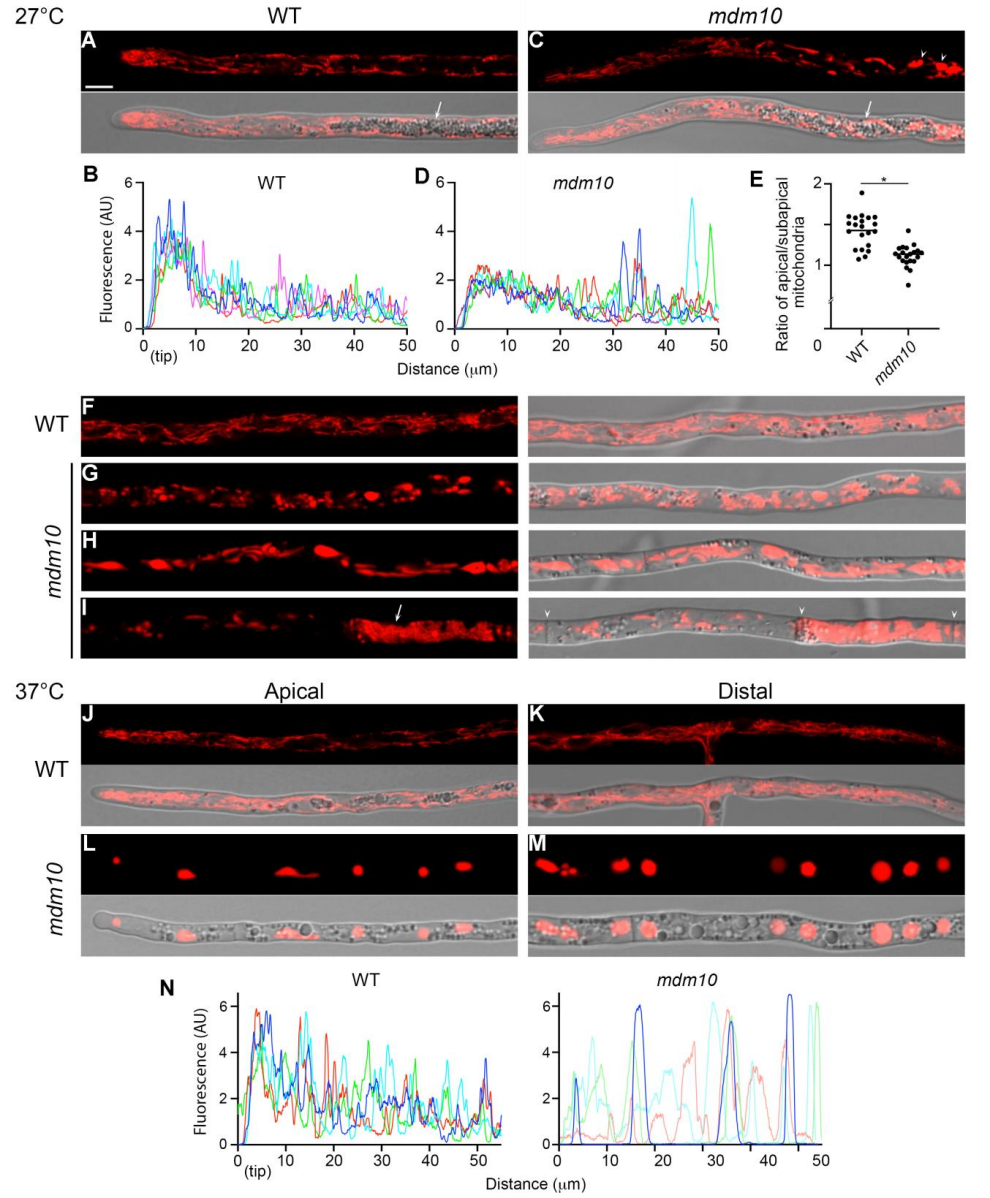
FIGURE 1: *P. anserina* mitochondrion arrangement and distribution depend on MDM10. **(A-D) **Localization of IDH1-mCherry-labeled mitochondria in the apical segment (i.e. the first ~80 μm behind the tip) of wild-type (WT) **(A)** and *mdm10-1* (*mdm10*) **(C)** leading hyphae growing at 27°C. Arrows indicate the region of high lipid droplet accumulation. Arrowheads point to large, round mitochondria. **(B, D)** Line scan graphs of IDH1-mCherry fluorescence intensity profile along the apical segment of wild-type **(B)** and *mdm10-1* **(D)** growing leading hyphae. Each line corresponds to an individual hypha. AU, Arbitrary units. **(E)** Ratio of the average mitochondrial IDH1-mCherry fluorescence between the apical (the first 10 µm from the tip) and subapical (10-30 µm) hyphal regions of wild-type and *mdm10-1* strains. Scatterplots show the mean of 21 hyphae issued from three independent experiments. *, P < 0.0001 by unpaired *t* test. **(F-I)** Localization of IDH1-mCherry-labeled mitochondria in subapical (F, G, behind 80 µm from the tip) and distal (H, I, beyond 150 µm from the tip) regions of wild-type and *mdm10-1* leading hyphae growing at 27°C. The arrow shows a network of tightly packed mitochondria, arrowheads point to septa. **(J-M)** Localization of mitochondria in the apical and distal regions of wild-type and *mdm10-1* leading hyphae growing at 37°C. Scale bar, 5 µm. **(N)** Line scan graphs of IDH1-mCherry fluorescence intensity profile along the apical segment of wild-type and *mdm10-1* leading hyphae growing at 37°C.

To investigate the role of the ERMES complex, we analyzed the function of MDM10 by studying thermosensitive *mdm10-1* (*mdm10^L420P^*) mutant strains growing either at permissive (27°C) or nonpermissive (37°C) temperatures. The initial analysis of mitochondria in this mutant performed by staining cells with DASPMI showed that their morphology was affected when growing at 27°C, resulting in a mix of enlarged and morphologically normal mitochondria 40. We extended these observations by analyzing mitochondria following the localization of an mCherry-tagged version of mitochondrial isocitrate dehydrogenase (IDH1-mCherry) expressed from its native locus 38. First, we found that, in addition to defective morphology, the distribution of mitochondria is affected when MDM10 is deficient. In wild-type leading hyphae (the hyphae at the extending edge of the colony), mitochondria consist of a dynamic network of elongated organelles with a high density accumulating at the apical region (i.e., in the first ~10 μm from the tip) (**Fig. 1A, B**), as described for diverse filamentous fungi (e.g., 41,42). In contrast, in the *mdm10-1* strain mitochondria failed to accumulate at the apical region to the same extent as in wild-type hyphae (**Fig. 1C, D**). Analysis of the ratio of apical (located in the first 10 µm from the tip) to subapical (between 10-30 µm from the tip) mitochondria showed a significant decrease in the proportion of mitochondria accumulating in the apical region of *mdm10-1* hyphae (**Fig. 1E**).

Next, we found that the impact of *mdm10-1* mutation on mitochondrial arrangement was distinct along different hyphal regions. We have shown that in *P. anserina* growing leading hyphae, the region extending ~40 μm behind the tip is followed by a region of about 50 μm where large "vacuolar" compartments accumulate (**Fig. 1A, C**, arrows), we show now that these organelles are lipid droplets (see below). In *mdm10-1* hyphae, the first 40 μm behind the tip showed mitochondria that were mostly elongated and exhibited a normal morphology (**Fig. 1C**). After this segment, mitochondria morphology mostly consisted of enlarged and spherical mitochondria (**Fig. 1C**, arrowheads, and **Fig. 1G**). Mito-chondria morphology was more severely affected towards the distal hyphal regions, which showed giant globose mitochondria (**Fig. 1H**), as well as densely packed networks of interwoven mitochondria (**Fig. 1I**, arrow). In addition, mitochondria distribution was also affected in distal hyphal regions, including hyphal compartments with low mitochondrial abundance. We observed that this defective distribution was associated with the aberrant mitochondrial morphology, which produced high local cytoplasmic crowding, as well as the occlusion of the septal pores that communicate hyphal compartments (**Fig. 1I**, and Mov. S1).

Finally, we also analyzed mitochondrial arrangement in hyphae growing at 37°C. In contrast to the wild-type, we found that* mdm10-1* hyphae contained mostly large and giant globose mitochondria throughout mycelia (**Fig. 1, J-N**), consistent with previous observations 40.

### Lipid droplets localize to the subapical hyphal region depending on MDM10

**Figure 2:**
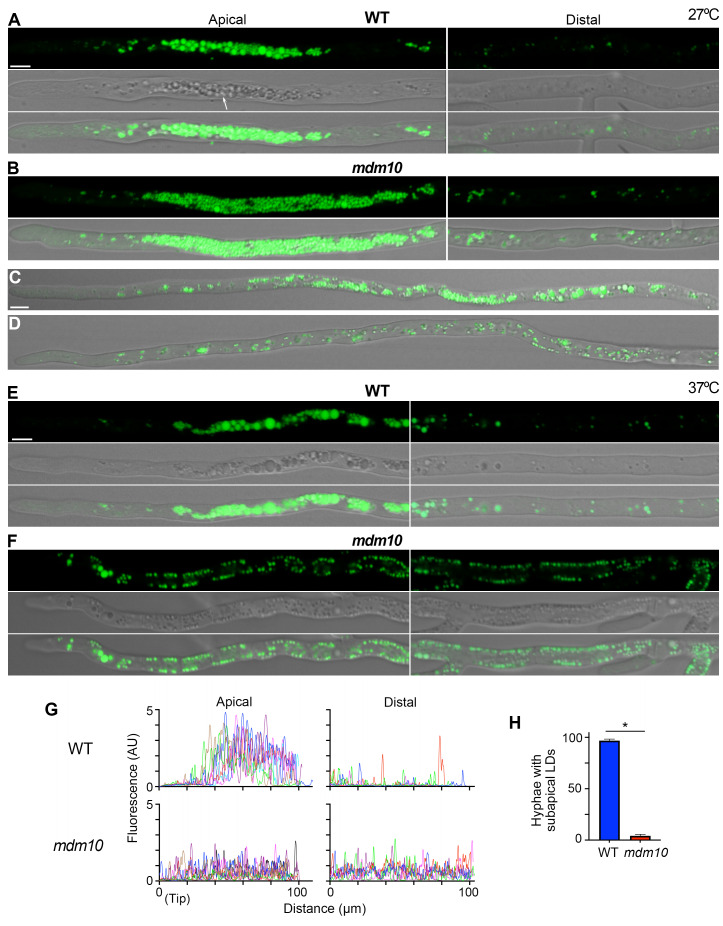
FIGURE 2: Lipid droplet localization depends on MDM10. **(A)** Localization of BODIPY 593/503-labeled lipid droplets in wild-type (WT) **(A)** or *mdm10-1* **(B-D)** leading hyphae growing at 27°C. The arrow point to the lipid-droplet accumulation region. **(B-D)** shows distinct lipid-droplet arrangements of *mdm10-1 *hyphae. Lipid droplet localization in wild-type **(E)** and *mdm10-1* **(F)** leading hyphae growing at 37°C. Left and right panels in (A, B, E and F) show the lipid droplet arrangement in the apical (the first ~100 μm behind the tip) and distal (~100-200 μm behind the tip) hyphal regions, respectively. Scale bar, 5 µm. At 27°C, lipid droplets accumulated in a region extending from 41.9 ± 10.7 (m to 91.5 ± 15.5 (m (avg. ± S.D., n=52) behind the tip in the wild-type, and between 41.1 ± 17.3 (m and 108.6 ± 36.2 (m (n=52) in *mdm10-1*. This region was in average larger in *mdm10-1 *(67.4 ± 33 (m) than in the wild-type (49.6 ± 15.2 (m) (*P*=0.0006 by unpaired *t* test). At 37°C, the wild-type lipid droplet accumulation region extended from 44.7 ± 14.3 to 110.3 ± 27.3 µm behind the tip (n=52¬¬). **(G)** Fluorescence intensity profile of BODIPY-labeled lipid droplets along the apical (left) and distal (right) regions of wild-type (top) and *mdm10-1* (bottom) leading hyphae growing at 37°C. Each line corresponds to an individual hypha. **(H)** Number of leading hyphae (per 100 hyphae) showing accumulation of lipid-droplets in the subapical region (LDs, lipid droplets). The histogram shows the mean ± S.D. of 6 biological replicates (100 hyphae/replicate: n=600 hyphae per strain). *, P < 0.0001 by unpaired *t* test.

We have described that *P. anserina* growing leading hyphae comprise a subapical region where large "vacuolar" compartments accumulate (in our standard dextrin-based growing media) (e.g. 38). However, we found that these organelles are not labeled by the vacuolar dye Oregon Green 488, or by FM4-64, which stains the compartments of the endocytic/vacuolar pathway (Fig. S1A and B). In contrast, these organelles were readily labeled by the neutral lipid dye BODIPY 493/503, which stains lipid droplets (**Fig. 2A**). In wild-type leading hyphae growing at 27°C, lipid droplets accumulated in a region starting about 40 (m behind the tip and extending for (50 (m (**Fig. 2A**, left). Lipid droplets were small and scarce behind this region (**Fig. 2A**, right). Lipid droplets also accumulated in the subapical region of most *mdm10-1* leading hypha growing at 27°C, however, this region was in average larger (67 (m) than in the wild-type (**Fig. 2B**), and its arrangement was more heterogeneous, as it consisted of clusters of densely packed lipid droplets (**Fig. 2B**), or with a scattered distribution (**Fig. 2C**). Moreover, a number of *mdm10-1* leading hypha (21.9%, n=155) lacked a distinctive regional accumulation of lipid droplets (**Fig. 2D**), a condition not observed in the wild-type.

Wild-type leading hyphae growing at 37°C also showed a region of high lipid-droplet accumulation (**Fig. 2E**). In contrast, this accumulation was not observed in most *mdm10-1* leading hyphae growing at this temperature (**Fig. 2F, G** quantitation in **H**); instead, these hyphae contained small lipid droplets dispersed along hyphae, even in distal hyphal regions (compare the apical and distal regions in **Figs. 2E-G**). Moreover, in contrast to the wild-type, lipid droplets predominantly localized to the periphery of* mdm10-1* hyphae, closely apposed to the cell surface (**Fig. 2F**). To explore whether the cortical localization of lipid droplets was produced by the disorganization of an additional organelle, such as excessive crowding of organelles like vacuoles, we analyzed the distribution of Oregon Green-stained vacuoles in *mdm10-1* hyphae. Similar to other filamentous fungi (e.g 43), vacuoles exhibit a differential distribution along *P. anserina* wild-type hyphae, where the apical hyphal region predominantly contains small rounded vacuoles, which are followed by a network of highly elongated and ramified vacuoles (shown for hyphae growing at 37°C in Fig. S1C). Large, rounded vacuoles accumulate beyond this region. However, we observed that *mdm10-1* hyphae exhibited a similar vacuolar arrangement to the wild-type, even when growing at 37°C (Fig. S1D). These results show that the localization of lipid droplets depends on MDM10.

### Peroxisome morphology and motility depend on MDM10

**Figure 3:**
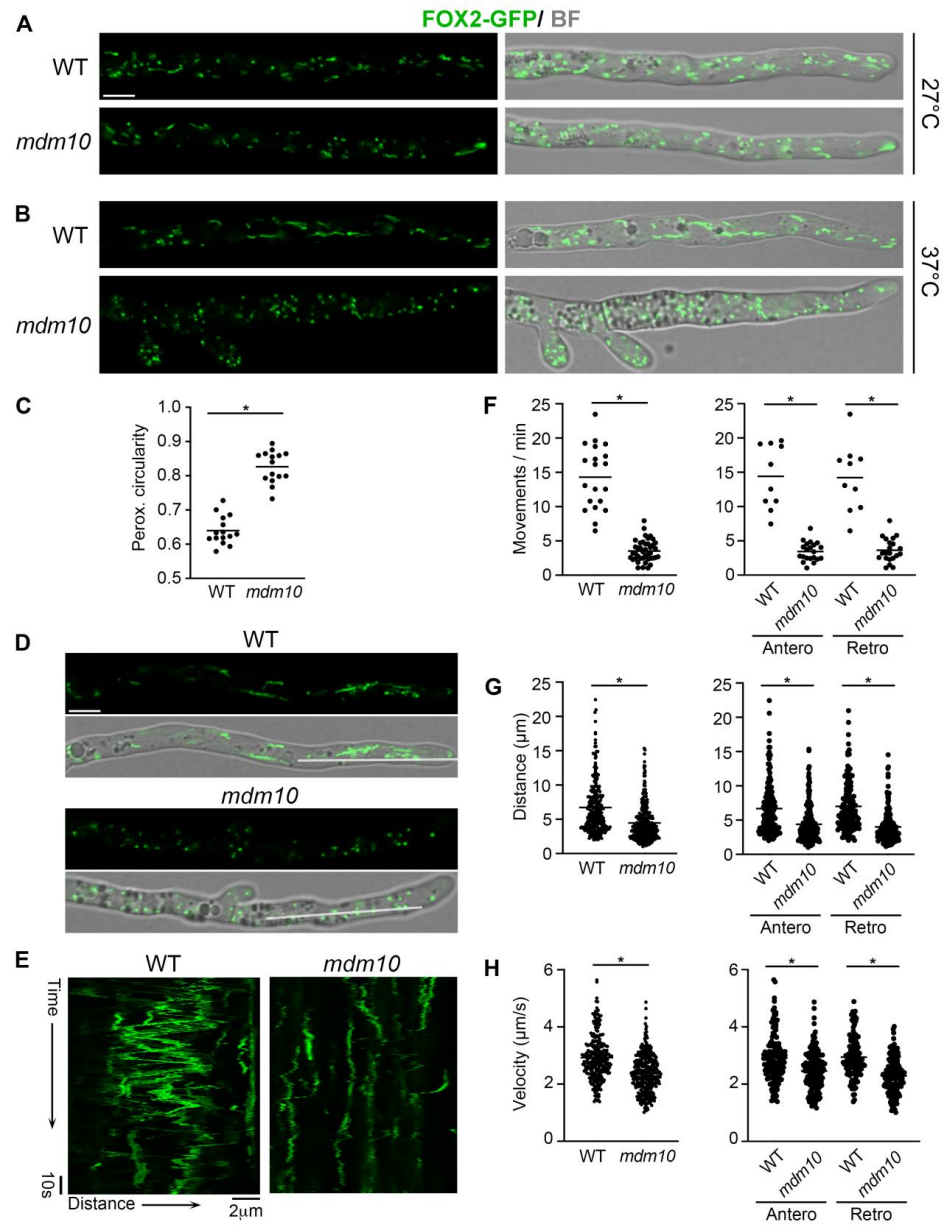
FIGURE 3: Peroxisome shape and motility depend on MDM10. Localization of FOX2-GFP-labeled peroxisomes in wild-type (WT) and *mdm10-1* leading hyphae growing at 27°C **(A)** or 37°C **(B)**. Scale bar, 5 µm. BF, Bright field. **(C)** Peroxisome circularity of wild-type and *mdm10-1* hyphae growing at 37°C. Data shows the average peroxisome circularity (a value of 1.0 corresponds to a perfect circle) of 15 hyphae issued from 3 experiments. *, P < 0.0001 by unpaired *t* test. **(D-E)** Still frames **(D)** of timelapse movies (see accompanying Movies S2 and S3) of wild-type and *mdm10-1* hyphae expressing FOX2-GFP growing at 37°C; **(E)** kymographs generated from the corresponding lines in **(D)**. **(F-H)** Quantitative analysis of peroxisome motility of wild-type and *mdm10-1* hyphae growing at 37°C. **(F)** Number of total (left), or anterograde and retrograde (right) peroxisome movements per minute per hyphae (normalized to a 100 µm^2^ area). n = 10 (WT) or 20 (*mdm10*). *, P < 0.0001 by unpaired *t* test (left) or one-way ANOVA with Tukey’s post hoc test (right). Distance **(G)** and velocity **(H)** of the overall (left), or anterograde and retrograde (right) peroxisome trajectories. n: WT = 273 peroxisome movements (147 anterograde/126 retrograde); *mdm10 = *310 (158 anterograde/152 retrograde) (issued from 6 and 8 hyphae, respectively). *, P < 0.0001 by unpaired *t* test (left) or one-way ANOVA with Tukey’s post hoc test (right).

Next, we studied the participation of MDM10 in peroxisome dynamics by analyzing the localization of an endogenously tagged version of the peroxisome fatty acid β-oxidation multifunctional protein 2 (FOX2-GFP) 38. First, we analyzed the arrangement of peroxisomes in hyphae growing at 27°C and we did not detect peroxisomal alterations in the *mdm10-1* cells, as compared to the wild-type (**Fig. 3A**). Then we analyzed peroxisome dynamics in hyphae growing at *mdm10-1* restrictive temperature (37°C). Under these conditions, wild-type peroxisomes are highly dynamic and predominantly adopt an elongated morphology. In contrast, *mdm10-1* hypha displayed mostly punctate spherical peroxisomes (**Fig. 3B**). Changes in peroxisome morphology were determined by analyzing peroxisome circularity, which was significantly higher in *mdm10-1* cells than in the wild-type (**Fig. 3C**). These data show that peroxisome membrane remodeling requires MDM10 activity.

We also observed that in wild-type hyphae growing at 37°C, peroxisomes are highly motile and exhibit frequent, fast, long-distance displacements both towards the tip (anterograde) and away from it (retrograde). We discovered that peroxisome motility was significantly reduced in *mdm10-1* hyphae (**Fig. 3D-E** and Movs. S2, S3). We found that the frequency of peroxisome movements was importantly reduced when MDM10 was deficient (**Fig. 3F**, left), and that the frequency of anterograde and retrograde movements was similarly affected in this mutant (**Fig 3F**, right). We also observed that the average distance of the trajectories of *mdm10-1* moving peroxisomes (4.5 µm) was shorter than in the wild-type (6.7 µm) (**Fig. 3G**, left), and that this defect was also similar for both anterograde (6.7 in wild-type *vs* 4.4 µm in *mdm10-1*) and retrograde (7.0 in wild-type *vs* 4.0 µm in *mdm10-1*) displacements (**Fig. 3G**, right). In addition, we observed a moderate reduction in the velocity of *mdm10-1* moving peroxisomes (avg. 2.4 µm/s), compared to the wild-type (2.9 µm/s) (**Fig. 3H**, left). Again, this defect was similar for anterograde (2.9 in wild-type *vs* 2.5 µm/s in *mdm10-1*) and retrograde (2.9 in wild-type *vs* 2.3 µm/s in *mdm10-1*) displacements (**Fig. 3H**, right). These data show that peroxisome motility also requires the activity of MDM10.

### ER organization depends on MDM10

**Figure 4:**
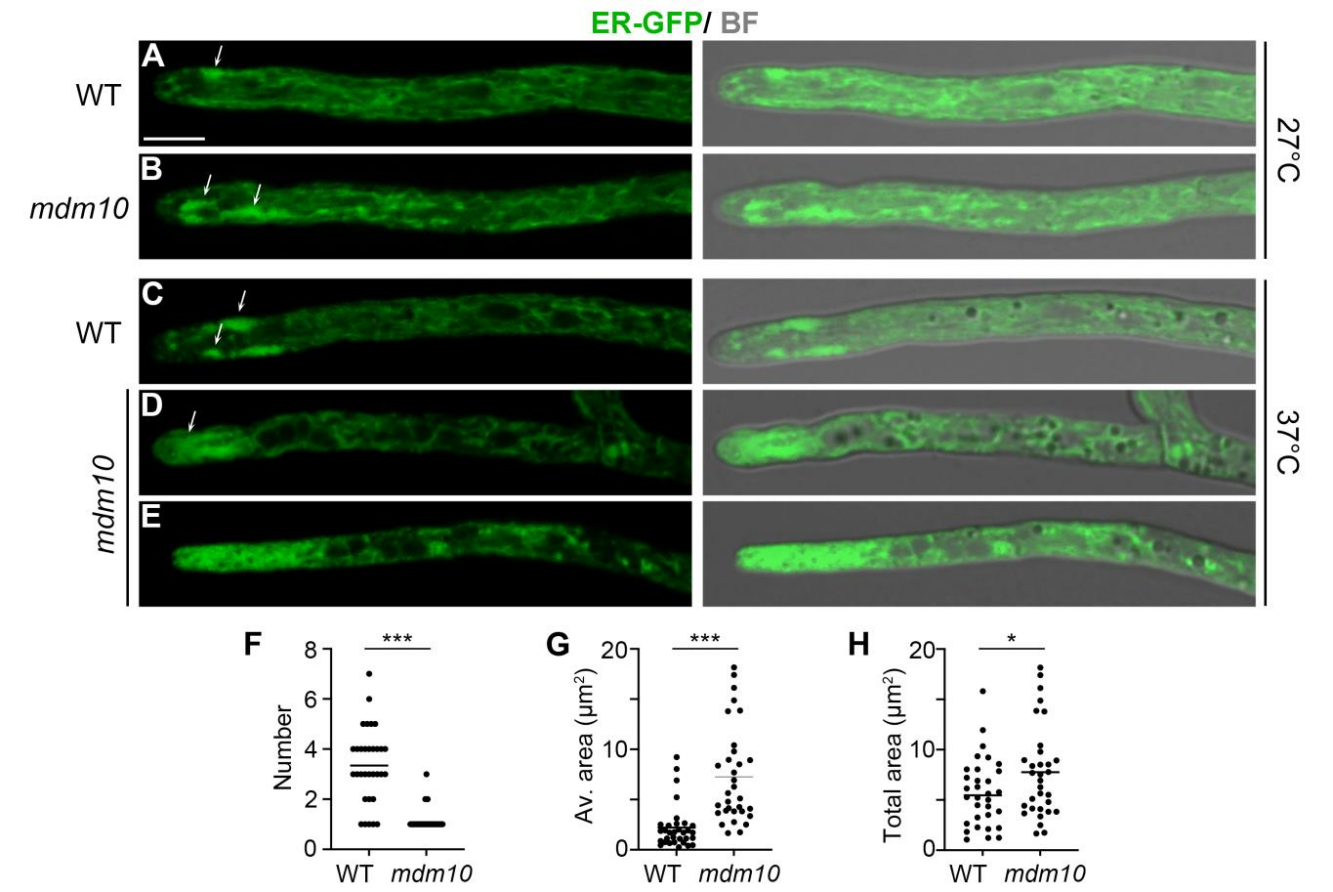
FIGURE 4: ER arrangement depends on MDM10. Localization of ER-GFP-labeled ER in wild-type (WT) and *mdm10-1* leading hyphae growing at 27°C **(A, B)** or 37°C **(C-E)**. Arrows indicate apical ER subcompartments. Scale bar, 5 µm. BF, Bright field. Scatterplots of the number **(F)**, average area **(G)** or total area **(H)** of the apical ER subcompartments per hyphae of wild-type and *mdm10-1* strains growing at 37°C. n = 32 (from 4 independent experiments). ***, P < 0.0001, *, P < 0.01 by unpaired *t* test.

Next, we asked whether MDM10 was also required for ER organization. First, we analyzed the localization of an ER-targeted GFP (ER-GFP), containing the ER targeting and retention sequences of the chaperone BiP. By studying this protein, we have shown that the *P. anserina* ER is composed of distinct peripheral ER domains, including a prominent apical domain, which consists of highly dynamic pleomorphic subcompartments (**Fig. 4A**, arrows) that are specifically associated with the apical region of growing hyphae (i.e., the first 15 µm behind the tip) 44. We studied the arrangement of the ER in *mdm10-1* and wild-type hyphae growing at permissive (27°C) (**Fig. 4A, B**) and restrictive (37°C) (**Fig. 4C-E**) temperatures, and we observed that the organization of the ER was importantly altered in *mdm10-1* cells growing at 37°C. At this temperature, wild-type leading hyphae contained numerous apical ER subcompartments (**Fig. 4C**), which were highly dynamic and underwent anterograde and retrograde displacements accompanied by fusion and division events. In contrast, the apical ER of *mdm10-1* frequently consisted of a single large subcompartment, or of a highly convoluted cluster of apical subcompartments (**Fig. 4D**). In some cells, the apical region of *mdm10-1* hyphae contained a densely packed amass of ER membranes (**Fig. 4E**). Quantitative analyses showed that the number of discrete subcompartments composing the apical ER in *mdm10-1* growing at 37°C was reduced, as compared to the wild-type (**Fig. 4F**), whereas their average (**Fig. 4G**) or total (**Fig. 4H**) area was increased.

**Figure 5:**
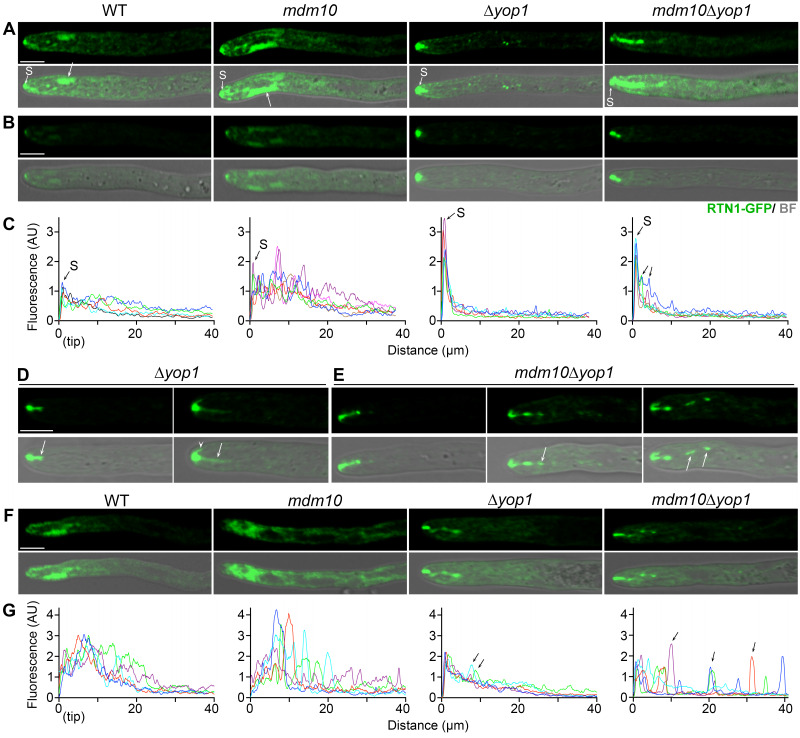
FIGURE 5: Apical ER organization requires MDM10 and YOP1. **(A, B)** Localization of RTN1-GFP in leading hyphae of the indicated strains growing at 27°C. Note that (A) and (B) were acquired with different imaging parameters, in (A) the detection was enhanced to better visualize the apical ER subcompartments (arrows), whereas in (B) it was adjusted to avoid signal saturation. *S*, Spitzenkörper. BF, Bright field. **(C)** Line scan graphs of RTN1-GFP fluorescence intensity profile along growing leading hyphae, as imaged in B. Each line represents an independent hypha. Arrows point to the signal associated to RTN1-GFP-labeled strands emerging from the Spitzenkörper. **(D, E) **Detail of the Spitzenkörper-associated localization of RTN1 in Δ*yop1 ***(D)** and *mdm10*Δ*yop1* **(E)** hyphae. Arrows point to RTN1-labeled strands emerging from the Spitzenkörper. **(F)** Localization of RTN1-GFP in leading hyphae of the indicated strains growing at 37°C. Scale bar, 5 µm. **(G)** RTN1-GFP fluorescence intensity profile along leading hyphae growing at 37°C, as in (F). Each line represents a hypha. WT: wild-type.

To further understand the contribution of MDM10 in apical ER organization, we studied the localization of an endogenously tagged version of the reticulon RTN1 39. Reticulons are ER membrane proteins that function in ER shaping and remodeling by promoting the formation of regions of high membrane curvature 45. In *P. anserina*, the single reticulon protein RTN1 localizes to the peripheral ER and accumulates in the apical ER domains (**Fig. 5A** and Mov. S4). In addition, RTN1 associates with the Spitzenkörper (*S* in **Fig. 5A**) 39, a hyphal apical body that orchestrates localized vesicle secretion at the hyphal tip to facilitate the local cell surface expansion that drives polarized hyphal growth 46. We analyzed RTN1-GFP localization in *mdm10-1* hyphae growing at 27°C and found that it localized to the peripheral ER (**Fig. 5A**, and Mov. S5), with a large accumulation in the apical ER domains (**Fig. 5A**, arrows). In addition, a fraction of RTN1 was associated to the Spitzenkörper (*S* in **Fig. 5A**). However, we observed that the RTN1-labeled apical ER domains were larger in *mdm10-1* hypha than in the wild-type, as revealed by analyzing the profile of RTN1-GFP fluorescence along hyphae (**Fig. 5A-C**).

By analyzing the localization of ER-GFP, we previously discovered that the formation of the apical ER subcompartments is reduced upon elimination of RTN1, and virtually abolished by eliminating YOP1 39, a second ER membrane curvature-generating protein involved in ER shaping and remodeling 45. Therefore, we asked whether the increase of the apical ER in *mdm10-1* cells involved YOP1 function. First, we analyzed RTN1-GFP localization in strains deleted for *YOP1* (Δ*yop1*), and we observed that the formation of the RTN1-labeled apical ER domains was virtually abolished (**Fig. 5 A, B**), as previous described for ER-GFP 39. We also observed that the signal of RTN1-GFP associated to the Spitzenkörper was concomitantly increased in these cells (**Fig. 5B, C**). Moreover, we observed the formation of short RTN1-labeled strands dynamically extending from the Spitzenkörper (Mov. S6), either backwards (**Fig. 5D**, arrows) or laterally subjacent to the cell surface (**Fig. 5D**, arrowhead). Some short segments separated from these strands while moving retrogradely and were seemingly incorporated into the peripheral ER. Eventually, a few short segments were redirected to the Spitzenkörper by fast anterograde displacements (Mov. S6). The precise nature of these Spitzenkörper-derived compartments remains to be established; however, in wild-type cells we observed a RTN1-labeled membrane flow stemming from the Spitzenkörper, which converged in the apical ER domains (Mov. S4). Therefore, the Δ*yop1* Spitzenkörper-derived elongated compartments could represent precursor membranes for the apical ER domains, which fail to properly structure in absence of YOP1.

Then we analyzed RTN1 localization in *mdm10-1*Δ*yop1 *double mutants and found that it was similar to that of Δ*yop1*. The formation of the apical ER domains was also virtually abolished in *mdm10-1*Δ*yop1*, with a concomitant increase in the Spitzenkörper-associated RTN1-GFP signal (**Fig. 5B, C**). We found that the RTN1-labeled strands extending from the Spitzenkörper in Δ*yop1* cells were also formed in *mdm10-1*Δ*yop1* (**Fig. 5A, E**, and Mov. S7); however, their formation was more profuse (**Fig. 5A, E**), and the segments stemming from them were more abundant (**Fig. 5C** and **E**, arrows, and Mov. S7). These observations show that the defects in the apical ER arrangement produced upon MDM10 dysfunction involve YOP1 function. Moreover, these findings also suggest that an Spitzenkörper-associated membrane traffic is involved in apical ER shaping, and that this process is influenced by MDM10 function. Of note, we also observed that the RTN1 membrane flow trafficking through the Spitzenkörper also occurred in *mdm10-1* hyphae, where it was ostensibly more profuse than in the wild type (compare Movs. S4 and S5).

Next, we analyzed the distribution of RTN1 at restrictive temperature and we observed that *mdm10-1 *mutation produced a disorganization of the peripheral ER similar to that revealed by ER-GFP, which included the formation of large apical ER domains, and the accumulation of ER membranes at the hyphal apical region (**Fig. 5F, G**). We also observed that RTN1-GFP localization in Δ*yop1 *hyphae was similar to that observed at 27°C, including the absence of apical ER domains and the formation of Spitzenkörper-derived elongated compartments (**Fig. 5F**). We found that RTN1 localization in *mdm10-1*Δ*yop1 *double mutants was similar to that of Δ*yop1 *(**Fig. 5F**), showing that the more dramatic disorganization of the apical ER produced by MDM10 deficiency at restrictive temperature also involves YOP1 function. In addition, we observed that the formation of RTN1-labeled strands extending from the Spitzenkörper was also more profuse in *mdm10-1*Δ*yop1* than in Δ*yop1* cells at 37°C (**Fig. 5G**).

### Early Golgi distribution is not affected by MDM10 dysfunction

The apical ER subdomains are specifically associated with the polarized growing hyphal region, but their actual role is unclear. In the basidiomycete *Ustilago maydis*, a large accumulation of motile ER compartments is also present in the apical hyphal region. This accumulation is dispersed upon treatment with brefeldin A -a drug that disturbs the ER to Golgi anterograde transport- suggesting that they are involved in ER-Golgi trafficking 47. To test whether the apical ER disorganization produced by MDM10 dysfunction affected Golgi organization, we analyzed early (cis) Golgi organization in the *mdm10-1* mutants. We generated strains expressing a GFP N-terminally tagged version of putative sorting receptor RER1, a cis-Golgi retrieval receptor for ER resident proteins 48, which effectively labels early Golgi in mycelial ascomycetes, like *Aspergillus nidulans* 49. Consistent with localization at the early Golgi cisternae, GFP-RER1 exhibited a punctate pattern with increased accumulation towards the apical region (Fig S2, A and B), similar to that described in *A. nidulans* 49. First, we asked whether RER1 was associated with the apical ER subdomains by comparing its localization to that of YOP2, a third ER curvature protein that also localizes to the apical ER 39. Although we detected some RER1-decorated cisternae persistently associated with the apical ER subcompartments (Fig. S2A, arrow), we did not observe colocalization between RER1- and the YOP2-decorated compartments, nor a predominant localization of the early Golgi cisternae in association with the apical ER domains (Fig. S2A). Next, we investigated if the organization of early Golgi cisternae was affected by the *mdm10-1* mutation. However, we did not detect major alterations in the arrangement of these compartments in *mdm10-1* cells growing either at 27°C or 37°C (Fig. S2B and C).

### MDM10 acts synergistically with YOP1 and RTN1

**Figure 6:**
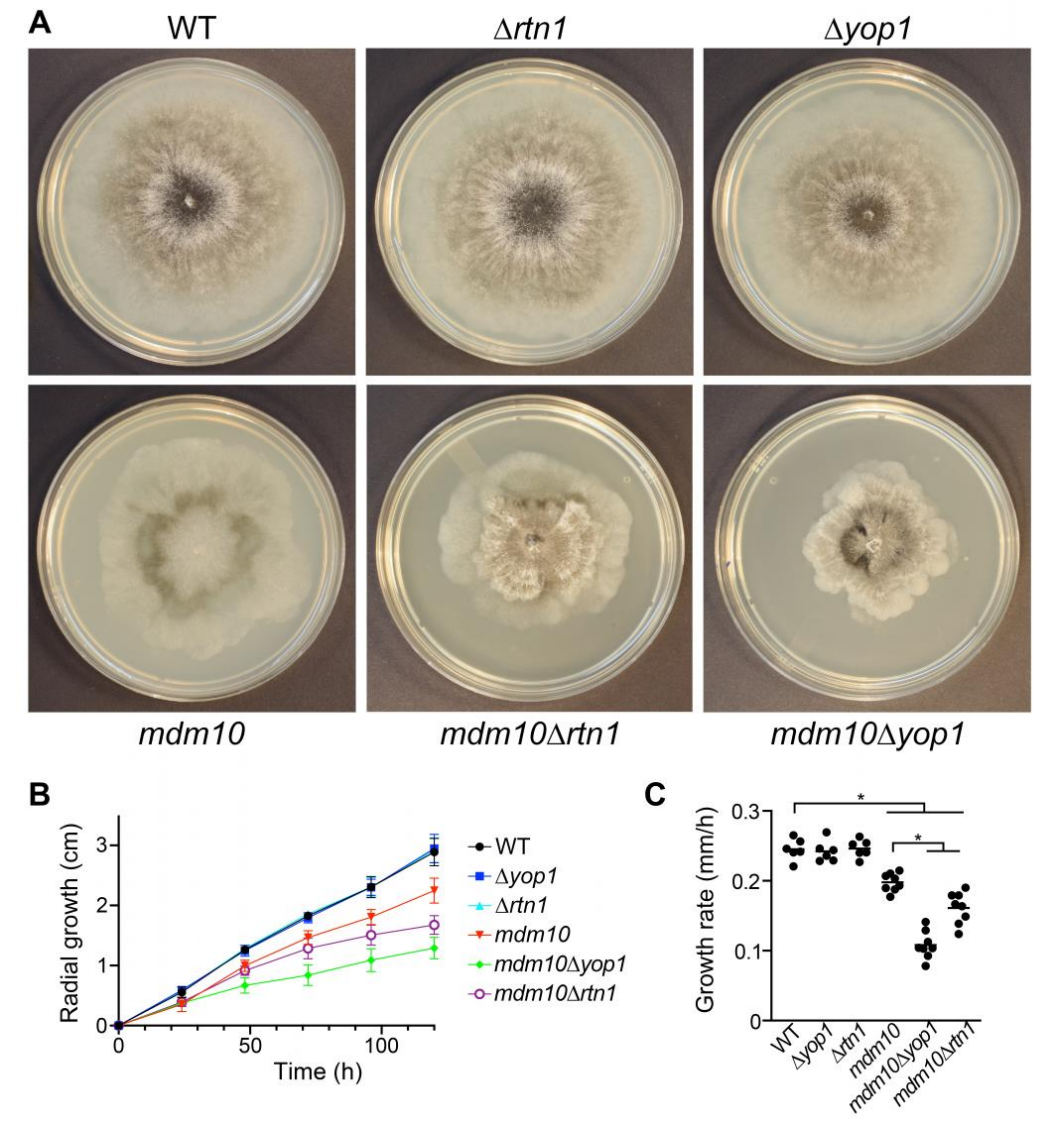
FIGURE 6: The mycelial growth defect of *mdm10-1* mutants is exacerbated by deleting *YOP1* or *RTN1*. **(A) **Mycelial colonies of the indicated strains grown for seven days at 27°C. Mycelial growth curves **(B)** and growth rates **(C)** of strains of the indicated genotypes. Data shows the average of three experiments, each with duplicates (n=6). *, P < 0.05 by one-way ANOVA with Tukey’s post hoc test. WT: wild-type.

To further address the relationship between MDM10 and the ER-shaping proteins, we analyzed the genetic interactions of *MDM10* with *YOP1 *and *RTN1*. Consistent with previous findings 40, we observed that *mdm10-1* mutation affects mycelial growth (**Fig. 6A-C**). We found that this defect was further enhanced when *mdm10-1 *mutation was combined with Δ*rtn1* or with Δ*yop1* (**Fig. 6 A-C**), which by themselves do not affect mycelial growth (**Fig. 6 A-C**) 39. These observations show that MDM10 and the ER-remodeling proteins YOP1 and RTN1 work together to control ER functioning, likely by regulating its shaping and interactions.

### The formation of sexual fruiting bodies is affected by MDM10 dysfunction 

**Figure 7:**
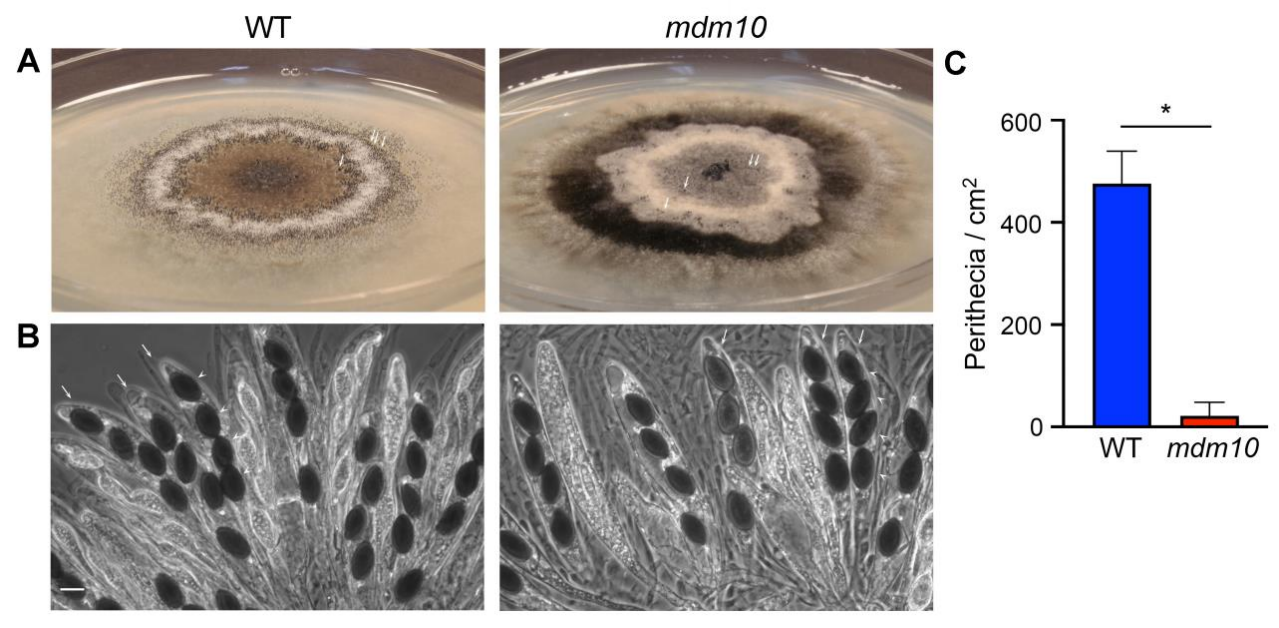
FIGURE 7: MDM10 dysfunction affects sexual development. **(A)** Homozygous sexual crosses of wild-type (WT) (left) and *mdm10-1* (right) strains (arrows point to perithecia). **(B)** Asci (arrows) bearing ascospores (arrowheads) issued from wild-type (left) and *mdm10-1* (right) homozygous crosses. Scale bar, 20 µm. **(C)** Number of perithecia produced in homozygous sexual crosses of the indicated strains. The histogram shows the mean ± S.D. of 4 independent colonies per strain. *, P = 0.0017 by unpaired *t* test.

We next studied MDM10 involvement in sexual development. *P. anserina* is a heterothallic fungus (possessing two distinct mating types, denoted as *MAT+* and *MAT-*) that reproduces exclusively sexually by forming multicellular fruiting bodies known as perithecia (**Fig. 7A**, arrows). Sexual development commences by the fertilization of a female gametangium (the ascogonium) by a uninucleate male gamete (the spermatium) of opposite mating type. The ascogonium is then surrounded by a tissue of tightly interwoven hyphae of maternal origin (i.e. emerging from vegetative hypha neighboring ascogonia), which develops into the perithecium envelope. Inside perithecia, the fertilized ascogonium produces the meiotic lineage where the sexual process occurs. This process begins with the differentiation from ascogonia of specialized dikaryotic cells, which undergo karyogamy and differentiate into asci, where meiosis occurs. The resulting meiotic nuclei then undergo mitosis to yield eight nuclei, which are subsequently isolated by pairs into four new cells produced within the ascus. These cells differentiate into ascospores (**Fig. 7B**), which are ultimately expelled out of perithecia. The spindle positioning during meiotic development and the high frequency of second meiotic-division segregation of the *MAT* locus result in ascospores containing two non-sister nuclei of opposite mating type, which give rise to self-fertile heterokaryotic strains. In a number of asci, one binucleated ascospore is replaced by two small uninucleate ascospores, which yield homokaryotic strains 50,51,52.

To analyze sexual development, we performed *mdm10-1* homozygous sexual crosses at 27°C, by using self-fertile dikaryotic strains that were homokaryotic for *mdm10-1* (i.e., *mdm10-1 MAT+ / mdm10-1 MAT-*). We found that *mdm10-1* strains were able to form perithecia and to produce ascospores; however, sexual development was notably affected (**Fig. 7A** and **B**). We observed that sexual development was delayed in *mdm10-1*, which produced perithecia seven days after inoculation, compared to four in the wild-type. Ascospore discharge from perithecia started six and nine days after inoculation for the wild-type and *mdm10-1* strains, respectively. In addition, we observed that the number of perithecia produced by *mdm10-1* was drastically reduced (**Fig. 7A** and **C**). These experiments showed that sexual development of *mdm10-1* mutants is affected; however, under these conditions, *mdm10-1* mutants were able to ultimately produce ascospores that were morphologically normal (**Fig. 7B**). *P. anserina* sexual reproduction does not take place at 37°C, which precluded analyzing sexual development at *mdm10-1* restrictive temperature.

### *MMM1* is required for vegetative growth

**Figure 8:**
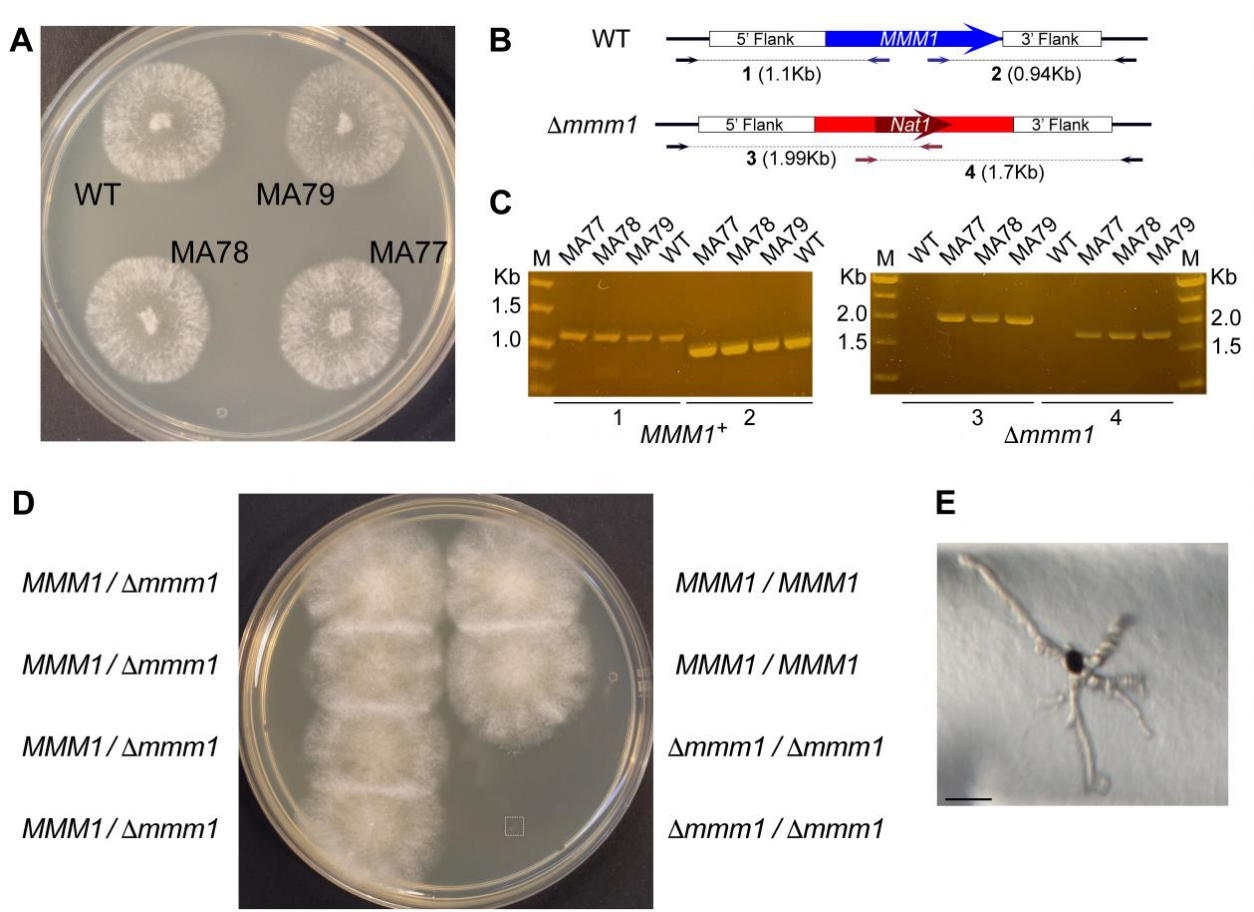
FIGURE 8: * MMM1* deletion is lethal. **(A)** Mycelial colonies of the wild-type (WT) and of heterokaryotic *MMM1/*Δ*mmm1* primary transformants (MA77-79). **(B-C)** Molecular characterization of *MMM1/*Δ*mmm1* primary transformants: **(B)** Schematics of the *MMM1* locus in the wild-type (top) and in Δ*mmm1* (bottom). 5’ and 3’ flanks indicate the ORF flanking regions used for the homologous recombination gene replacement. Arrows indicate primers used to detect the *MMM1 *and Δ*mmm1 *alleles by PCR, the expected sizes of the corresponding amplified products (discontinuous lines) are indicated below. **(C)** Confirmation of the heterokaryotic nature of the *MMM1/*Δ*mmm1* primary transformants by PCR, showing the amplification of *MMM1* (left) and Δ*mmm1* (right) alleles. M, molecular weight marker. **(D)** Tetrad analysis of the meiotic progeny of *MMM1/*Δ*mmm1* dikaryotic (self-fertile) strains. *P. anserina* meiotic development results in asci with four ascospores each containing two nuclei. For a given gene (here *MMM1*), segregation at first meiotic division results in two homokaryotic spores for each allele (right), while second meiotic division segregation produces four heterokaryotic spores (left). In this meiotic progeny, second division segregation resulted in four viable *MMM1/*Δ*mmm1* heterokaryotic ascospores (left), while first division segregation produced two homokaryotic *MMM1/MMM1* ascospores (right, top) and two homokaryotic Δ*mmm1*/Δ*mmm1* spores, which were unable to grow (right, bottom). **(E)** Magnification of the boxed area in **(D)**, showing the germination phenotype of a Δ*mmm1*/Δ*mmm1* homokaryotic spore. Bar, 50 µm.

To better analyze the role of the ERMES complex in sexual development we thus opted to delete *MDM10*. In addition, we decided to delete *MMM1*, which codes for the ER protein of ERMES. We elected to delete these genes by replacing their respective open reading frame (ORF) by the *Streptomyces noursei nat1* gene, which confers nourseothricin resistance. Despite numerous efforts, we were unable to recover nourseothricin-resistant (NatR) colonies when attempting to delete *MDM10*; however, we obtained three NatR colonies when deleting *MMM1 *(**Fig. 8A**). PCR analyses of these strains showed that they harbored both, a wild-type and a deleted allele (Δ*mmm1*) of the *MMM1* gene (**Fig. 8B** and **C**), consistent with a heterokaryotic (*MMM1/*Δ*mmm1*) genotype. By crossing these strains with the wild-type, we were able to recover the Δ*mmm1* allele in heterokaryotic strains issued from dikaryotic ascospores (i.e.,* MMM1/*Δ*mmm1*). These strains were able to self-fertilize and produced normal ascospores. The analyses of the meiotic progeny of these strains showed that the Δ*mmm1* allele could only be recovered in heterokaryotic (*MMM1/*Δ*mmm1*) ascospores, resulting from segregation at second meiotic division (**Fig. 8D**, left). We were not able to obtain Δ*mmm1* homokaryotic strains issued from dikaryotic ascospores produced upon first meiotic division segregation (**Fig. 8D**, right) or from uninucleate ascospores. Detailed inspection of the Δ*mmm1* homokaryotic ascospores showed that they were able to germinate and produced few short germ tubes; however, they ceased to growth early after gemination (**Fig. 8E**), even after supplementing the germination medium with yeast extract or after transferring the colonies to standard (M2) growth media. These findings show that MMM1 is strictly required for vegetative growth in *P. anserina*.

### MMM1 is required for sexual development

Next, we asked whether MMM1 was also required during the *P. anserina* sexual phase. To address this issue, we generated trikaryotic strains containing two Δ*mmm1* nuclei of opposite mating type, along with a nucleus deleted for the entire *MAT* locus (i.e., Δ*mmm1 MAT+/*Δ*mmm1 MAT-/MMM1*Δ*mat, *hereafter referred to as Δ*mmm1/*Δ*mat* trikaryon). The *MAT* locus contains the genes that govern sexual reproduction in fungi. In *P. anserina*, the *MAT* locus is strictly required for mating but has no discernible functions in vegetative cells 53. Thus, in these trikaryotic strains, the Δ*mat* nucleus provides a wild-type *MMM1* allele -which can rescue Δ*mmm1 *vegetative defects- but it cannot participate in sexual development (i.e. it cannot participate in fertilization events, thus, only Δ*mmm1 MAT+ *and Δ*mmm1 MAT- *nuclei can engage in sexual reproduction). The efficiency of generation of Δ*mmm1/*Δ*mat* trikaryotic strains (see Material and Methods) was very low; therefore, we also generated a Δ*mmm1MAT+/MMM1*Δ*mat* dikaryotic strain, to be tested as female partner in sexual crosses against a dikaryotic Δ*mmm1MAT-/MMM1 MAT+* strain (i.e. where Δ*mmm1 MAT+* can only be fertilized by Δ*mmm1 MAT-*). The outcome of both strategies is the potential generation within perithecia of a Δ*mmm1 *homozygous meiotic lineage.

**Figure 9:**
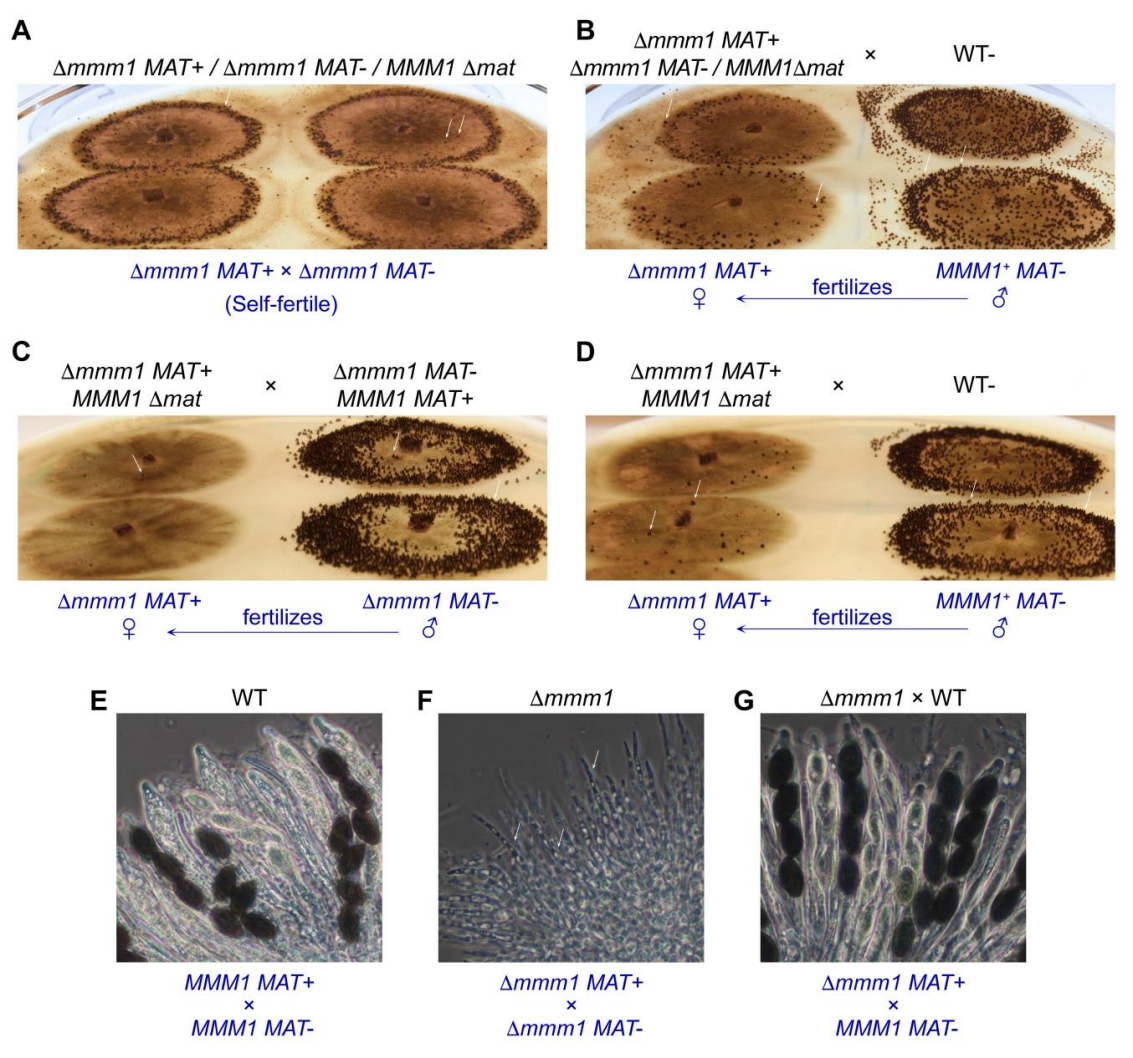
FIGURE 9: MMM1 is required for sexual development. Sexual cross of a Δ*mmm1MAT+/*(*mmm1MAT-/MMM1^+^*(*mat* trikaryotic strain self-fertilized **(A)**, or fertilized by wild-type (WT) spermatia **(B)**. The lower headings (blue) indicate the nuclei involved in the relevant fertilization events to produce the (*mmm1* homozygous **(A)** or heterozygous **(B)** meiotic lineages. Note that additional fertilization events can occur, for example, the WT colonies in (B, right) can be fertilized by (*mmm1MAT+* spermatia from the trikaryon. Sexual cross of a (*mmm1MAT+/MMM1*(*mat* dikaryotic strain to a (*mmm1MAT-/MMM1MAT+ *dikaryon **(C**, here only the (*mmm1MAT+ *gametangia of the (*mmm1MAT+/MMM1*(*mat* dykarion (left) can be fertilized, and only by the (*mmm1MAT-* spermatia of (*mmm1MAT-/MMM1MAT+*), or to a wild-type strain (**D**, (*mmm1MAT+ *gametangia can be fertilized by WT spermatia). Arrows point to perithecia. **(E-G)** Sexual cycle cells issued from a wild-type homozygous cross **(E)**, from a (*mmm1MAT+/*(*mmm1MAT-/MMM1^+^*(*mat* trikaryotic self-fertile cross (**F**, as depicted in A), or from a (*mmm1MAT+/MMM1*(*mat* dikaryotic strain acting as female fertilized by wild-type spermatia (**G**, as depicted in D, left colonies). The lower headings (blue) indicate the nuclei (potentially) involved in the meiotic lineages of these crosses. Arrows point to perithecia.

We obtained a Δ*mmm1/*Δ*mat* trikaryotic strain and we observed that it was able to produce perithecia (**Fig. 9A**). We also found that this strain was fertile in crosses against the wild-type (**Fig. 9B**) acting either as female (left) or male (right) partner. Perithecia produced when the Δ*mmm1/*Δ*mat* trikaryon self-fertilized (**Fig. 9A**) were smaller than in the crosses with the wild-type (**Fig. 9B**), and they were less abundant when the Δ*mmm1/*Δ*mat* trikaryon acted as female partner (**Fig. 9B** left, compare to right). We also obtained a Δ*mmm1MAT+/MMM1*Δ*mat* dikaryotic strain (hereafter referred to as Δ*mmm1/*Δ*mat* dikaryon), which was crossed with a Δ*mmm1 MAT-/MMM1 MAT+* heterokaryon. In this cross, the Δ*mmm1/*Δ*mat* dikaryotic strain produced very few fruiting bodies when acting as female partner, which only rarely developed into perithecia (**Fig. 9C**, arrow). Larger perithecia were produced by this strain when it was fertilized by wild-type spermatia, albeit in reduced numbers (**Fig. 9D**, left).

We found that for both types of crosses homozygous for Δ*mmm1* -the self-fertile Δ*mmm1/*Δ*mat* trikaryotic strain (**Fig. 9A**) or the Δ*mmm1/*Δ*mat* dikaryon crossed as female to a Δ*mmm1 MAT-/MMM1 MAT+* dikaryon (**Fig. 9C**, left)- the perithecia produced were sterile and were unable to produce asci and ascospores (shown for the Δ*mmm1/*Δ*mat* trikaryon in **Fig. 9F**, compare to **E**). We were also unable to detect ascogenous hyphae (croziers) within these perithecia, which contained only supportive vegetative hyphae (i.e., paraphyses: sterile vegetative hyphae of maternal origin present within perithecia, arrows in **Fig. 9F**). Both, Δ*mmm1/*Δ*mat* dikaryotic and trikaryotic strains were able to produce ascospores in sexual crosses to the wild-type, showing that their sterility phenotype is recessive (shown for the Δ*mmm1/*Δ*mat* dikaryon in **Fig. 9G**). These findings show that MMM1 is strictly required for *P. anserina* sexual development.

## DISCUSSION

The ERMES complex constitutes a main mitochondrion-ER tethering complex in fungi. This complex has an important function in the transfer of lipids between these organelles, and plays multiple roles that are critical for mitochondrial functioning. The ERMES complex also participates in the regulation of the dynamics of additional organelles, like peroxisome proliferation. Here we demonstrate that in *P. anserina* the ERMES complex is required for the dynamics of mitochondria, lipid droplets, peroxisomes and the ER. Moreover, we show that the ERMES complex is essential for* P. anserina* growth and development.

Along with the initial characterization of *P. anserina* MDM10 40, we found that MDM10 dysfunction produces defects in mitochondrial morphology, which include the formation of large spherical mitochondria and of densely packaged mitochondrial networks. In *P. anserina*, these mitochondrial morphologies are also produced when the mitochondrial fission proteins DNM1 or FIS1 are missing 38, showing that the elimination of mitochondria-ER contacts and of mitochondrial fission produces common outcomes. We also found that MDM10 is required for mitochondrial distribution, and that the effect of MDM10 dysfunction on mitochondrial morphology differs along distinct hyphal regions, producing stronger defects in subapical/distal regions. Again, this same phenomenon was observed in *P. anserina* by disrupting the mitochondrial fission machinery 38. These observations indicate that mitochondria regulation is subject to distinct regional hyphal constrains, and could indicate the existence of mechanisms that ensure the localization of high-functioning mitochondria at the places of polarized hyphal growth.

We also discovered that in *P. anserina* the ERMES complex is required for peroxisome shaping and motility. First, we found that MDM10 is required for peroxisome elongation. Peroxisome elongation is associated to specific physiological conditions, such as temperature and oxidative stress 37, and could reflect a stage of peroxisome proliferation. In *Saccharomyces cerevisiae*, the ERMES complex is involved in peroxisome proliferation 30,31,54; however, in this yeast the ERMES complex appears to negatively regulate this process. In *P. anserina*, MDM10 could be involved in transducing the physiological cue that induces peroxisome elongation. Alternatively, MDM10 could participate in peroxisome membrane remodeling. In line with this interpretation, in *S. cerevisiae* Mdm34 physically interacts with Pex11 31, which is the key peroxin implicated in peroxisome membrane elongation 55,56,57,58, and this peroxin contributes to the peroxisomal alterations produced by ERMES deficiencies 54. Interestingly, in mammalian cells PEX11 induces peroxisome membrane protrusions that interact with mitochondria, suggesting that peroxisome elongation might facilitate the interactions between these organelles 59. In yeasts, peroxisomes localize in proximity to ERMES sites, often in close association with the ER 14,30, supporting the existence of three-way junctions between mitochondria, peroxisomes and the ER. Contacts between peroxisomes and the ER are required for peroxisome membrane expansion 60,61; therefore, MDM10 could also contribute to peroxisome membrane expansion by promoting ER-peroxisome associations. The ERMES complex could also be involved in peroxisome remodeling by acting on the mitochondrial Rho-GTPase Miro, which in yeasts (Gem1) associates with the ERMES complex 62,63. Miro proteins localize to mitochondria and peroxisomes and regulate their dynamics 64. In mammalian cells, Miro proteins promote peroxisome elongation by negatively regulating the activity of the fission GTPase Drp1 (Dnm1) 65.

We also found that MDM10 is required for peroxisome motility. In mycelial fungi, peroxisomes move by hitchhiking on early endosomes, which are transported by microtubule-based motor proteins 66,67. As illustrated by the ER-endosome interaction, the formation of membrane contact sites between organelles can modulate their association to motor proteins, and thereby regulate their motility 68,69. It is tempting to speculate that ERMES-mediated organelle interactions are required to promote peroxisome transport by regulating the transfer of motility regulatory proteins, such as the adapters that associate peroxisomes and endosomes (or their motor proteins). The ERMES complex could also be involved in peroxisome motility by acting on the Miro GTPases, which in mammalians act as motor adaptors for mitochondria 64 and are required for proper peroxisome motility 65,70,71.

We also found that lipid droplets exhibit a specific subapical hyphal localization in *P. anserina*, which depends on the ERMES complex. The significance and the mechanism responsible for this accumulation are currently unknown. However, lipid droplet and peroxisome motility depend on similar transport systems in *U. maydis* 66, although this might differ among fungi 72. Lipid droplets originate from the ER 73, and they can form from ER domains that are involved in peroxisome biogenesis 74,75 or that interact with mitochondria 76,77. Lipid droplets maintain continuous associations with the ER, as well as extensive dynamic interactions with mitochondria and peroxisomes 78,79,80. The hyphal localization of lipid droplets could depend on (some of) these interactions, which could be disrupted by the defects in organelle dynamics produced by ERMES dysfunction. Alternatively, the ERMES complex could directly mediate associations between lipid droplets and mitochondria or the ER. In line with this hypothesis, Mmm1 and Mdm34 interact with the lipid droplet protein Erg6 in *S. cerevisiae *81. Interestingly, in addition to altered localization along hypha, the ERMES dysfunction also produced a relocalization of lipid droplets to the cell periphery. It is tempting to speculate that when the ERMES-dependent associations are lost, lipid droplets predominantly interact with the cortical ER or with the plasma membrane.

We discovered that *P. anserina* MDM10 is also required for ER shaping. In contrast, the elimination of ERMES proteins in *S. cerevisiae* does not notably alter ER morphology 26,82. We found that the organization of the peripheral apical ER domains, which are associated with the polarized growing hyphal region, depends on MDM10. Moreover, we found that the effect of MDM10 dysfunction in ER shaping involves the ER-shaping protein YOP1 -which is required for the formation of the apical ER subcompartments 39-, and we discovered that the combined mutations of *YOP1* and *MDM10* cause a synthetic growth defect. Likewise, we observed that the growth defect of *mdm10-1* is enhanced by eliminating RTN1, a second ER-shaping protein involved in apical ER organization 39. Altogether, these observations indicate that the ERMES complex is involved in ER shaping, and that its activity is functionally associated with that of YOP1 and RTN1. Consistent with our findings, loss of Yop1 and Rtn1 in *S. cerevisiae* produces a synthetic growth defect when combined with the elimination of any ERMES component 83. Moreover, our findings also suggest that the function of the apical ER domains and of mitochondria are associated.

Our research also disclosed an association between ER remodeling and the vesicle trafficking system that conducts polarized hyphal growth, which is influenced by ERMES activity. Polar hyphal growth depends on the Spitzenkörper, a dynamic apical structure that collects secretory vesicles near the tip, to be later delivered to the plasma membrane for polarized cell surface growth 46. In *P. anserina*, RTN1 localizes to the apical ER domains and to the Sptizenkörper 39. We found that loss of the apical ER domains produced by eliminating YOP1 results in increased association of RTN1 to the Spitzenkörper, and in the formation of elongated compartments extending from the Spitzenkörper. These compartments could reflect a Spitzenkörper-associated membrane traffic involved in apical ER shaping. The increased formation of these compartments produced by MDM10 dysfunction suggests that the organization of this membrane traffic is influenced by the contacts that the ER forms with mitochondria.

Our research discovered that *MMM1* is strictly required during the somatic phase of *P. anserina*, suggesting that it is essential. Our inability to obtain *MDM10 *deletion strains suggests that this gene could also be essential. Consistent with our findings, research in *Aspergillus fumigatus* 84 and in *A. nidulans* 85 has shown that ERMES complex proteins are also required for mycelial growth in these fungi. However, contrary to these findings, mycelial growth in *Neurospora crassa* was not notably affected by *mdm10* deletion, and it was only reduced by* mmm1* deletion 86. These observations suggest that different systems have evolved in fungi to regulate organelle interactions. In addition, we demonstrated that the formation of sexual fructifications depends on MDM10, and that MMM1 is also strictly required for sexual development*. *In keeping with this finding, *mmm1* mutation in *N. crassa* produces female sterility 87. Altogether, these findings show that the ERMES complex perform multiple roles in the regulation of organelle functioning in *P. anserina*, with critical growth and developmental impacts.

## MATERIALS AND METHODS

### Strains, plasmids and culture media

The strains of *P. anserina* used in this research were derived from the *S* wild-type background. *P. anserina* ∆*ku70* strain 88 was used for the gene deletions. *mdm10-1* 40 and ∆*mat* 53 strains were kindly provided by Veronique Contamine and Robert Debuchy, respectively. *S. noursei nat1* gene was derived from plasmid pAPI509, a derivative of pAPI508 88. Plasmid pCCG::N-GFP 89 was used for tagging of RER1 with GFP. *P. anserina* growth and sexual reproduction medium consisted of M2 minimal medium with 1.1% dextrin as carbon source. Ascospores were germinated on G medium supplemented with 0.5% yeast extract, and protoplasts were regenerated on RG medium. Live cell imaging was performed on MM medium (M2 containing 0.55% dextrin, and 2% agarose replacing agar). Media were supplemented with nourseothricin (40 mg mL-1), phleomycin (40 mg mL-1) or hygromycin B (75 mg mL-1) when required. Media composition can be consulted at http://podospora.i2bc.paris-saclay.fr

### Nucleic acid isolation, transformation and gene deletions

*P. anserina* DNA isolation and transformation were performed according to 90. *MMM1 *gene was deleted by replacing its ORF with the nourseothricin-resistance gene *nat1* by homologous recombination. The gene replacement cassette was generated by double-joint PCR and consisted of *nat1* gene flanked by (700 bp of the 5′ and 3′ *MMM1* ORF flanking sequences. The PCR fragments were obtained with primers: *mmm1-5F* and *mmm1-5R* (*MMM1* 5’ flank), *mmm1-nourF* and *mmm1-nourR* (*nat1*), and *mmm1-3F* and *mmm1-3R* (*MMM1* 3’ flank). The final fusion PCR product was gel-purified and used to transform ∆*ku70* protoplasts. Nourseothricin-resistant (*Nat^R^*) transformants were recovered and analyzed by PCR using primers: *mmm1-5Fc*, *mmm1-OR*, *mmm1-3Rc*, *mmm1-OF*, *Nour-F* and *Nour-R* (Supp. Table 1 and **Fig. 8B**). All recovered strains were heterokaryotic (*MMM1*/Δ*mmm1*). The strains were then crossed with the wild-type, and purified heterokaryotic *MMM1*/Δ*mmm1* strains in the *KU70^+^* genetic background were recovered. Multiple attempts to delete *MDM10* gene by the same strategy (i.e., using a gene replacement construct generated with primers: *mdm10-5F*, *mdm10-5R*, *mdm10-3F*, *mdm10-3R*, *mdm10-nourF* and *mdm10-nourF*) were unsuccessful. Oligonucleotide primer sequences can be consulted in Supp. Table 1.

### Tagging of RER1 

We generated strains ectopically expressing a N-terminally GFP-tagged version of RER1 (Pa_7_10200, XP_001908387) under the control of the *N. crassa* *ccg-1* promoter. Double-joint PCR was used to fuse a PCR fragment containing *GFP* gene preceded by the *ccg-1* promoter (amplified from plasmid pCCG::N-GFP using primers *CCG1-F* and *rer-GFP-R*) to the coding sequence of *RER1* gene followed by 658 bp of its 3’ flanking sequence (amplified with primers *GFP-rer1-F* and Rer*1-3Ra*). The obtained fusion PCR product was cloned into plasmid pGEM-t Easy (Promega, Madison, WI), was verified by sequencing and was used to co-transform wild-type cells along with the plasmid pSM334_Genticin. Randomly selected geneticin-resistant transformants were crossed to the wild-type, and the purified *GFP::RER1* transgene was recovered in homokaryotic strains.

### Microscopy 

Confocal microscopy was done on a Zeiss LSM800 inverted laser scanning confocal microscope equipped with a controlled environment chamber using Plan-Apochromat 63x/1.4 or 40x/1.3 oil immersion objectives and laser lines 488 and 561 nm. Bright-field images were obtained with an Electronically Switchable Illumination and Detection (ESID) module, and multi-channel imaging was done by collecting all channels simultaneously. Live cell imaging was done under controlled temperature, at 27°C or 37°C. Images were processed using ZEN 3.9 (Carl Zeiss, Jena, Germany) or ImageJ (FIJI) (NIH, Bethesda, Maryland) software 91,92.

### Cytological and quantitative analyses

Live cell imaging was done on whole individual *P. anserina* colonies grown for 24 h on MM at 27°C as described previously 93. For the experiments at restrictive temperature, colonies were first incubated at 27°C for 12-18 h, and then for another 12 h at 37°C. Peroxisomes, mitochondria and the ER were visualized following fluorescent tagged versions of FOX2, IDH1 and RTN1, respectively, expressed from their endogenous loci 38,39. To visualize the ER we also used an mCherry C-terminally-tagged version of YOP2, expressed from its native locus 39, as well as an ER lumen-targeted GFP (ER-GFP), consisting of GFP flanked by the ER targeting and retention sequences of the ER chaperone BiP 44. Vacuoles were labeled with Oregon Green 488 Carboxylic Acid Diacetate (carboxi-DFFDA SE) (Molecular Probes) (1.5 µM), the endocytic/vacuolar pathway compartments with FM4-64 [N-(3-triethylammoniumpropyl)-4-(6-(4-(diethylamino) phenyl) hexatrienyl) pyridinium dibromide] (7 µM) (Molecular Probes), and lipid droplets with BODIPY 493/503 (4,4-Difluoro-1,3,5,7,8-pentamethyl-4-bora-3a,4a-diaza-s-indacene) (Sigma-Aldrich) (1 µM). The analyses of mitochondria and ER distribution, peroxisome circularity, and the line scan graph analyses were done on confocal micrographs of the mid-plane of the apical segment of growing hypha ((70-100 µm in length). To quantify the ratio of apical/subapical mitochondria, the average IDH1-mCherry fluorescence intensity (defined as the mean gray value) of the apical (10 µm from the tip) and subapical (between 10 and 30 µm from the tip) segments of a hypha were calculated on ImageJ. For each strain, 21 hyphae issued from 3 biological replicates were analyzed. To analyze peroxisome circularity, FOX2-GFP confocal images were thresholded and the peroxisome circularity was determined using the Particles analyzer in ImageJ. For each strain, 15 hyphae issued from 3 biological replicates were analyzed. Overall, 1045 WT and 1379 *mdm10* peroxisomes were analyzed. Apical ER abundance was quantified by measuring the area occupied by the apical ER patches in thresholded ER-GFP confocal images using the ImageJ Particles analyzer. For each strain, 32 hyphae issued from 4 biological replicates were analyzed. Statistical significance was determined by unpaired t-tests. Peroxisome motility was analyzed on confocal time-lapse series of growing leading hyphae expressing FOX2-GFP imaged at 205-348 ms intervals for 1-2 min. Motility statistics were determined using the MTrackJ Plugin 94 of ImageJ. The frequency of peroxisome movements was calculated on 1min-long time-lapse series (n=10 for the wild-type, 20 for *mdm10-1*). The distance and velocity of peroxisome movements was calculated with 273 wild-type and 310 *mdm10-1* peroxisome trajectories issued from 6 and 8 hyphae, respectively (each issued from an independent strain). Statistical significance was determined by unpaired t-test or one-way ANOVA with Tukey’s multiple comparisons test. Peroxisome kymographs were generated using KymographBuilder in ImageJ (FIJI). Perithecia number was quantified in wild-type and *mdm10-1* homozygous sexual crosses at 27°C by using self-fertile dikaryotic strains (i.e., [*MDM10 MAT+/MDM10 MAT-*]* and *[*mdm10-1 MAT+/mdm10-1 MAT-*]*, *respectively). To quantify perithecia number, the perithecia present in a 1/16 radial sector of a colony was counted under the stereoscope 6 and 9 days after inoculation, respectively. For each strain, 4 independent colonies were quantified, and for each colony, 3 different sectors were counted.

### Generation of Δ*mmm1* dikaryotic and trikaryotic strains.

To obtain Δ*mmm1 MAT+/*Δ*mmm1 MAT-/MMM1*Δ*mat* trikaryotic strains, we took advantage of the incipient germination of Δ*mmm1* ascospores. We collected newly germinated Δ*mmm1MAT+/*Δ*mmm1MAT-* dikaryotic ascospores (i.e., homokaryotic for Δ*mmm1*) issued from first division segregation and mix them with fragmented Δ*mat* mycelium. The mycelial mix was overlaid on nourseothricin-containing media and NatR colonies were recovered. We generated a Δ*mmm1MAT+/MMM1*Δ*mat* dikaryotic strain by the same strategy: we collected Δ*mmm1 *homokaryotic germlings issued from uninucleate ascospores and mix them with fragmented Δ*mat* mycelium to recover NatR colonies.

## CONFLICT OF INTEREST

The authors declare no conflict of interest.

## SUPPLEMENTAL MATERIAL

Click here for supplemental data file.

Click here for supplemental data file.

Click here for supplemental data file.

Click here for supplemental data file.

Click here for supplemental data file.

Click here for supplemental data file.

Click here for supplemental data file.

Click here for supplemental data file.

All supplemental data for this article are available online at www.microbialcell.com/researcharticles/2025a-alvarez-sanchez-microbial-cell/.
