# Supplementary material for: Organelle activity organized by the endoplasmic reticulum-mitochondria encounter structure -ERMES- is essential for Podospora anserina development
Source: Microb Cell. 2025 Sep 12;12:255–73. doi: 10.15698/mic2025.09.860 (PMC12427119; doi:10.15698/mic2025.09.860)
Supplement: Supplementary file 1 [file mic-12-255-s01.pdf]

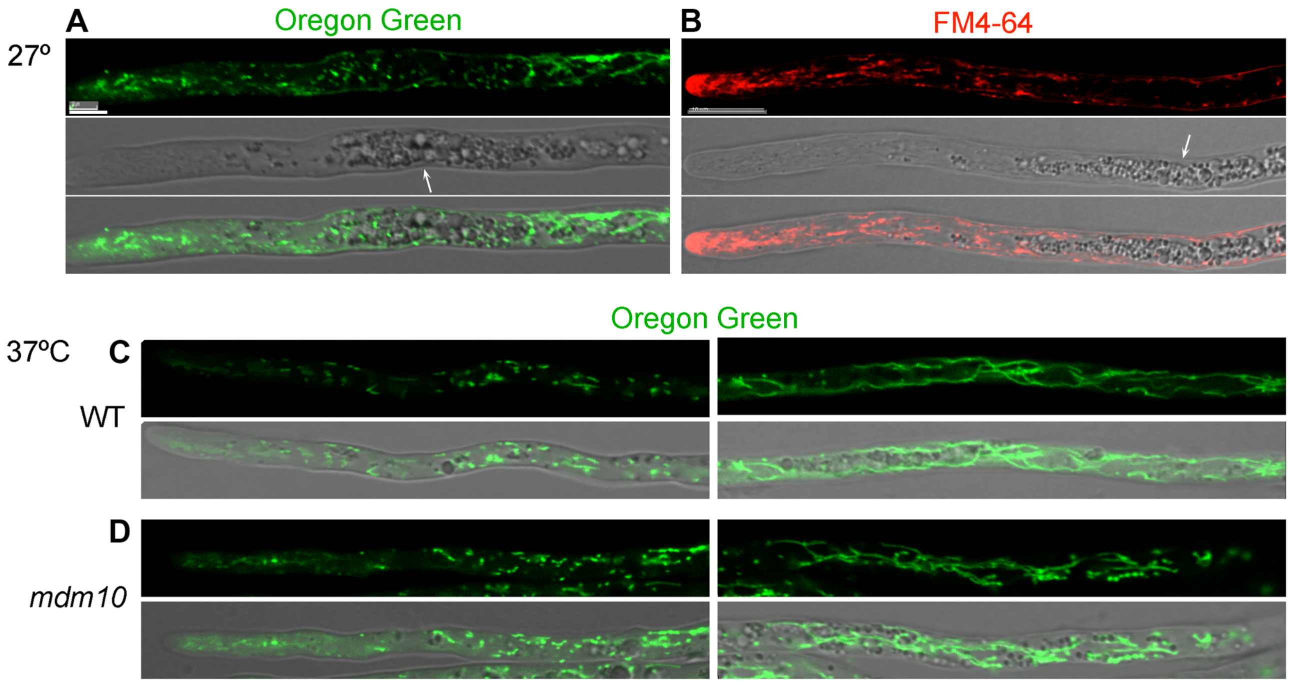

Figure S1. Arrangement of vacuolar/endocytic-compartments in *P. anserina* hyphae. Wild-type leading hyphae growing at 27°C stained with the vacuolar dye Oregon Green 488 (A) or with the vacuolar/endocytic compartment dye FM4-64 (B). Arrows point to the lipid-droplet accumulation region. Localization of Oregon Green-stained vacuoles in the apical (~80µm from the tip, left) and subapical (behind ~80µm, right) hyphal regions of wild-type (C) and *mdm10-1* (D) leading hyphae growing at 37ºC. Scale bar, 5 µm.

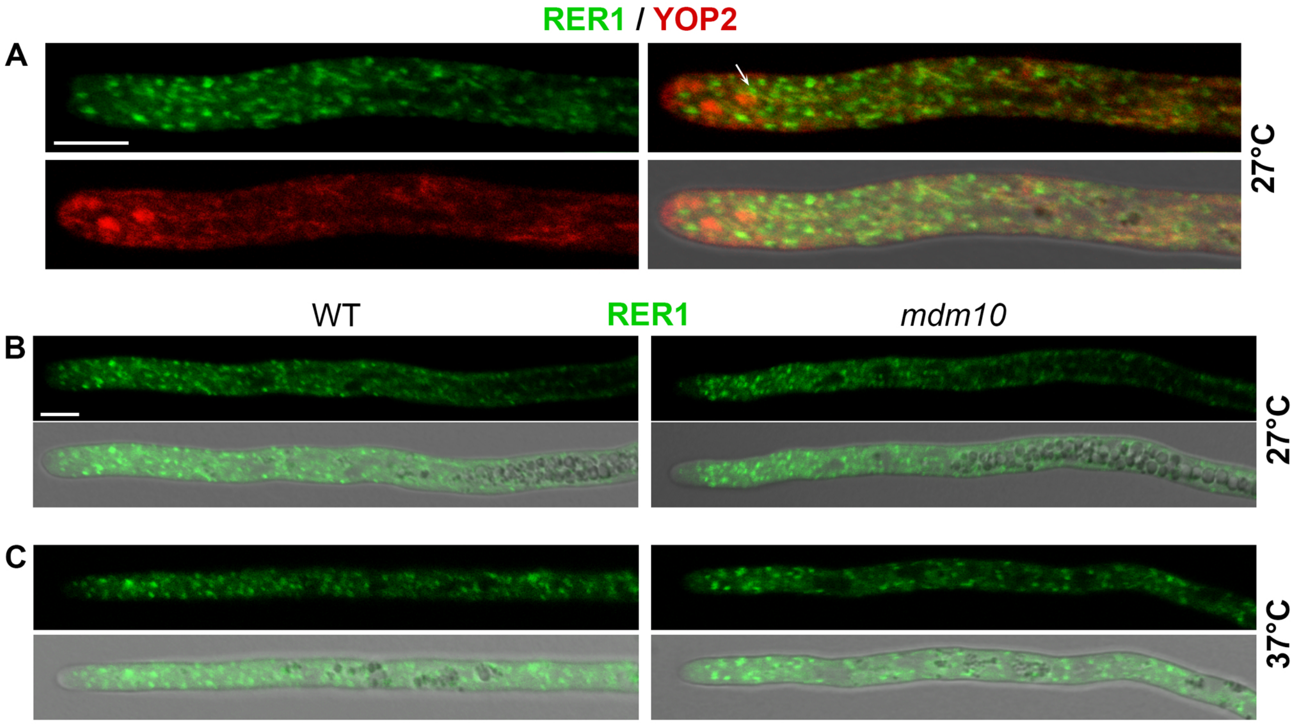

Figure S2. Localization of early Golgi cisternae in wild-type and *mdm10-1* hyphae. (A) Compared localization of GFP-RER1-stained early Golgi and YOP2-mCherry-labeled ER in wild-type leading hyphae growing at 27°C. Arrow points to an early Golgi cisterna in close proximity to an apical ER subcompartment. Localization of GFP-RER1-labeled early Golgi in wild-type (left) and *mdm10-1* (right) leading hyphae growing at 27ºC (B) or 37ºC (C). Scale bar, 5 µm.

Supplementary Table 1. Oligonucleotide primers used in this research.

Primer name	Primer sequence 5'>3'
mmm1-5F	GGTACAGGGGCTGAGACATGTTGAG
mmm1-5R	GAAGCATTTATCAGGGTCGCTGCTCAAGAAATTCG
mmm1-nourF	CGAATTTCTTGAGCAGCGACCCTGATAAATGCTTC
mmm1-nourR	CGGGACTTTGGAGATGAGCACGGAAATGTTGAATAC
mmm1-3F	GTATTCAACATTTCCGTGCTCATCTCCAAAGTCCCG
mmm1-3R	ACGCGCGTTTATGAAGGGTATCATG
mmm1-5Fc	TAGCAGGACAGCAATGAG
mmm1-OR	GCTTCGCCAAAGATGAAG
mmm1-3Rc	CGGCTAACCAAACGAATG
mmm1-OF	AACTTGACACTGAGACGG
Nour-F	CGACATCTCATCTTCCTG
Nour-R	GTTGACGTTGGTGACCT
mdm10-5F	GATTTCGGACAAGTCAGTGTTGGGC
mdm10-5R	GAAGCATTTATCAGGGTCTCCTCAATGCAGACCTG
mdm10-nourF	CAGGTCTGCATTGAGGAGACCCTGATAAATGCTTC
mdm10-nourR	CATTCCTCGTGGGCATTACACGGAAATGTTGAATAC
mdm10-3F	GTATTCAACATTTCCGTGTAATGCCCACGAGGAATG
mdm10-3R	AAGCGCAACTACCACTACTGCAGAG
CCG1-F	ATCGAATTCGTTCAAAGCCACATCA
rer-GFP-R	TGGTTCCTCGACAGCGTCGTTAATTAATGGCGCGCCG
GFP-rer1-F	CGGCGCGCCATTAATTAACGACGCTGTCGAGGAACCA
Rer1-3Ra	GCCGGATCCGTTGTGGTCCTGGTTGTCGAG
